# Comparative Genomic Analysis of Pathogenic and Probiotic *Enterococcus faecalis* Isolates, and Their Transcriptional Responses to Growth in Human Urine

**DOI:** 10.1371/journal.pone.0012489

**Published:** 2010-08-31

**Authors:** Heidi C. Vebø, Margrete Solheim, Lars Snipen, Ingolf F. Nes, Dag A. Brede

**Affiliations:** 1 Laboratory of Microbial Gene Technology and Food Microbiology, Department of Chemistry, Biotechnology and Food Science, The Norwegian University of Life Sciences, Ås, Norway; 2 Section for Biostatistics, Department of Chemistry, Biotechnology and Food Science, The Norwegian University of Life Sciences, Ås, Norway; Charité, Campus Benjamin Franklin, Germany

## Abstract

Urinary tract infection (UTI) is the most common infection caused by enterococci, and *Enterococcus faecalis* accounts for the majority of enterococcal infections. Although a number of virulence related traits have been established, no comprehensive genomic or transcriptomic studies have been conducted to investigate how to distinguish pathogenic from non-pathogenic *E. faecalis* in their ability to cause UTI. In order to identify potential genetic traits or gene regulatory features that distinguish pathogenic from non-pathogenic *E. faecalis* with respect to UTI, we have performed comparative genomic analysis, and investigated growth capacity and transcriptome profiling in human urine *in vitro*. Six strains of different origins were cultivated and all grew readily in human urine. The three strains chosen for transcriptional analysis showed an overall similar response with respect to energy and nitrogen metabolism, stress mechanism, cell envelope modifications, and trace metal acquisition. Our results suggest that citrate and aspartate are significant for growth of *E. faecalis* in human urine, and manganese appear to be a limiting factor. The majority of virulence factors were either not differentially regulated or down-regulated. Notably, a significant up-regulation of genes involved in biofilm formation was observed. Strains from different origins have similar capacity to grow in human urine. The overall similar transcriptional responses between the two pathogenic and the probiotic strain suggest that the pathogenic potential of a certain *E. faecalis* strain may to a great extent be determined by presence of fitness and virulence factors, rather than the level of expression of such traits.

## Introduction

Once considered as harmless commensals of the intestinal tract, enterococci now rank among the leading causes of infections among hospital patients [1], [2]. *Enterococcus faecalis* is among the most prevalent agents isolated from nosocomial urinary tract infections (UTIs), and is a common cause of chronic and recurrent UTIs, especially those associated with structural abnormalities and medical devices, such as urinary catheters [3]. The ability of *E. faecalis* to cause infection has been linked to inherent enterococcal traits, enabling the bacterium to tolerate harsh and diverse environments. In addition, several factors that may contribute to enterococcal virulence have been characterized (reviewed in [4]), and the role of these factors in pathogenicity have been further established in various animal models [5], [6], [7], [8] and cultured cell lines [9], [10]. However, a widespread distribution of putative virulence determinants in enterococcal isolates independent of origin has been reported [11], [12], [13], [14], [15], [16], and to date, no single virulence factor has been demonstrated to be essential for enterococcal infections. The ability of *E. faecalis* to cause infection is therefore likely to involve an orchestrated interplay between the regulation of these putative virulence factors and various genetic determinants that govern adaptation of the bacterial cell physiology during the infection process. Cultivation in urine partly mimics the urinary tract environment, and identification of differentially expressed genes *in vitro* may therefore represent a potential means to identify novel fitness factors required for this particular ecological niche.

Shepard and Gilmore previously examined the effect of growth in urine on the expression of known and suspected enterococcal virulence factors by quantitative real-time PCR [17], and significant changes in *E. faecalis* virulence-associated gene expression were observed in response to the biological cues present in urine, compared to laboratory medium-growth. Furthermore, studies of other pathogens causing UTI have reported responses involving iron acquisition systems and genes involved in sugar and amino acid metabolism [18], [19], which may indicate that bacteria suffer from glucose and iron limitation during growth in human urine.

In this report, we compare the global expression profiles of three *E. faecalis* strains during growth in human urine *in vitro*. The three strains were chosen based on their origins; the Symbioflor 1 strain, included in a commercial probiotic product used for more than fifty years without any reports of infection [20], the hospital outbreak strain MMH594 holding most known virulence genes in its genetic repertoire [21], [22], and finally the laboratory strain OG1RF which harbors some important virulence traits like *fsr* and *epa*, but is devoid of mobile genetic elements (MGEs) [23], [24]. This latter strain is however capable of causing infection in *e.g.* mice [23], [25], and has been extensively used as a model organism to investigate virulence ([4] and references therein). The aim of this work was to gain insight into genetic factors that make *E. faecalis* such a potent cause of human UTI. The study was designed to identify traits that distinguish pathogenic from non-pathogenic *E. faecalis*. Identification of such traits may ultimately contribute to development of strategies for prevention and treatment of *E. faecalis* UTI.

## Results and Discussion

### Growth capacity of different *E. faecalis* strains in urine and 2xYT

**Figure 1:**
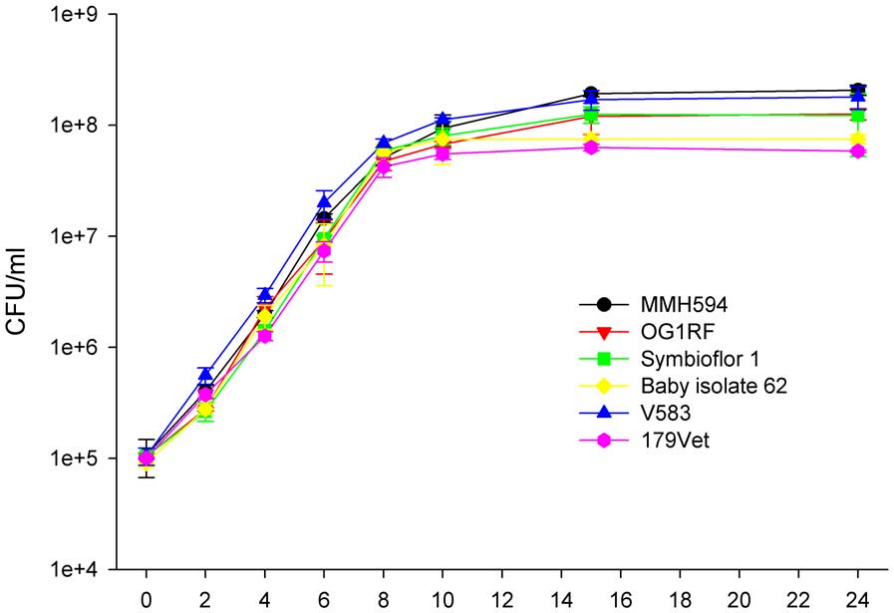
Growth of *E. faecalis* in urine. Characterization of growth of *E. faecalis* MMH594 (black circle), OG1RF (red triangle), Symbioflor 1 (green square), Baby isolate 62 (yellow diamond), V583 (blue triangle) and 179Vet (pink hexagon) in urine. The growth curves are represented by colony forming units per millilitre (CFU/ml) on the Y-axis, and hours as indicated on the X-axis. The growth curves correspond to the mean ± STD of two parallels.

*Escherichia coli* associated with UTI normally grow well in urine, while non-uropathogenic strains do not [26]. To examine whether this also could be true for *E. faecalis*, six strains of nosocomial, UTI, commensal or probiotic origin were cultivated in urine and colony forming unit (CFU) counts performed (Figure 1). Only minor differences in growth capacity were observed between the various isolates, with generation times of around 48 minutes (doubling time of 48.6±3.7 min). MMH594 and V583 reached a slightly higher final cell density (∼2.0×10^8^ CFU/ml) compared to OG1RF and Symbioflor 1 (∼1.2×10^8^ CFU/ml), and even more so compared to Baby isolate 62 and 179Vet (∼6.5×10^7^ CFU/ml). These observations are consistent with a recent study by Carlos *et al.* [27], where strains from diverse origins, such as food and clinical strains, did not grow significantly different in urine. Furthermore, the growth capacity of MMH594 observed in the present study was in agreement with previous reports [17].

Since the initial growth experiments did not reveal any strains with a distinctively enhanced or reduced growth capacity in urine, two pathogenic strains MMH594 and OG1RF, and the probiotic strain Symbioflor 1 were selected for further investigation by comparative genomic analysis and transcriptional analysis.

### Comparative genomic analysis

**Figure 2:**
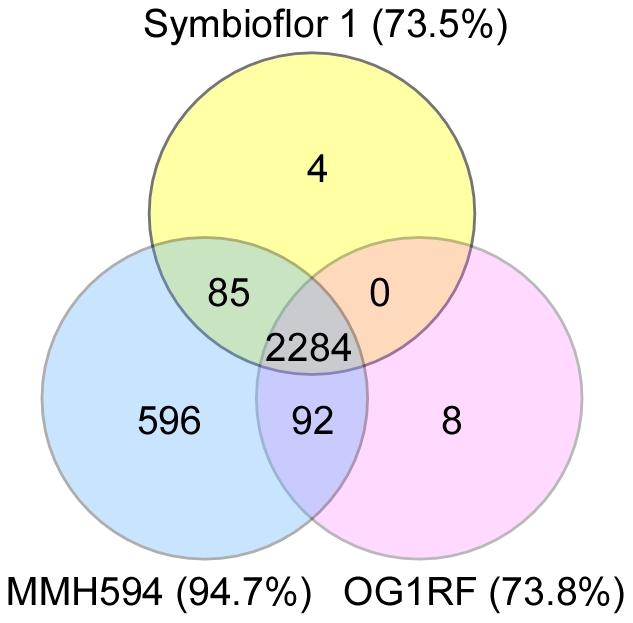
Gene content of *E. faecalis* MMH594, OG1RF and Symbioflor 1. Venn diagram showing the distribution of genes classified as present in the three test strains. The percentages indicated for each strain specify how large part of total probe set represented on the array that was classified as present in the corresponding strain.

**Figure 3:**
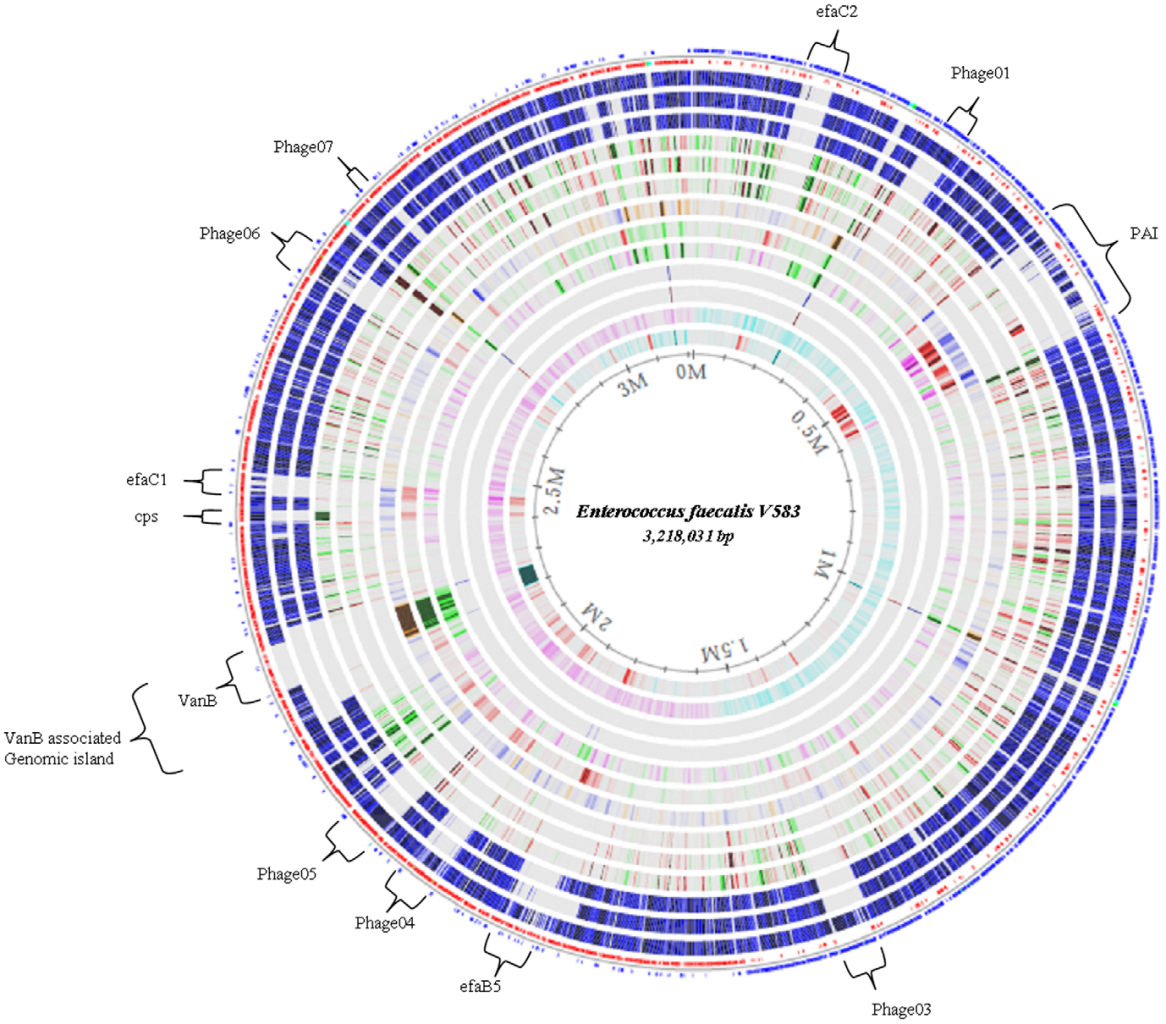
Genome-atlas presentation of CGH analysis and transcriptional responses to growth (t_30_) in urine compared to 2xYT. Mobile genetic elements [28], [31] are indicated by brackets. From outer to inner lanes: 1) V583 annotated CDS, 2) CGH MMH594, 3) CGH Symbioflor1, 4) CGH OG1RF, 5) Urine transcriptome MMH594, 6) Urine transcriptome Symbioflor1, 7) Urine transcriptome OG1RF, 8) Intrinsic curvature, 9) Stacking energy, 10) Position Preference, 11) Global direct repeats, 12) Global inverted repeats, 13) GC skew, 14) AT percent. Interactive Genewiz atlases of CGH and transcriptome data are available at; http://ws.cbs.dtu.dk/cgi-bin/gwBrowser/edit.cgi?hexkey=603430a081eb5be3f306e744e94b151a, and http://ws.cbs.dtu.dk/cgi-bin/gwBrowser/edit.cgi?hexkey=3068b894fb6b9e44d9c135a210950f52, respectively.

A comparative genomic analysis was conducted with emphasis on features that distinguish the three strains. The genomes of the strains used in the present study have previously been analyzed, and many aspects of the genomic composition have thus been accounted for (MMH594: [22], [28]; OG1RF: [23], [29]; Symbioflor 1: [20]. However, there are no sequence data publicly available for the Symbioflor 1 strain. Moreover, there existed no publicly available complete annotation for OG1RF. Thus, in order to obtain a detailed account of genetic variation and to validate the performance of our microarray, CGH was performed on the three strains (Figure 2, Figure 3 and Table S1). A total of 2284 genes were classified as present in all the strains tested. Not surprisingly, the clinical bacteremia isolate MMH594 showed the highest similarity to the reference strain V583 (94.7% genes in common). The presence of the entire pathogenicity island (PAI) in MMH594 was also confirmed [22], [30]. For the two other test stains the similarity to the reference strain was significantly lower, with 2384 (74.1%) and 2371 (73.7%) of V583 genes represented on the array classified as present in OG1RF and Symbioflor 1, respectively.

Altogether, MMH594 contains 596 genes that appear to be divergent in OG1RF and Symbioflor 1. Major variations in the presence of all the previously defined mobile genetic elements (MGEs) [28], [31] were observed between the three test strains. Except for *phage01* and *vanB*, all the MGEs seemed to be present in MMH594. *phage02* appear to be part of the *E. faecalis* core genome, while none of the other elements were found in OG1RF. This observation is consistent with the genome sequence available for OG1RF [23]. Symbioflor 1 contained certain genes/modules from *phage06*, but not the entire element. The rest of the MGEs were divergent by CGH in Symbioflor 1, which is consistent with previous reports [20]. Notably, Symbioflor 1 contains two major deletions in proximity to the *vanB* associated island and the efaB5 element (Figure 3 and Table S1). The latter deletion extends in the 5′ direction of efaB5 to EF1811 including the *fsr*-*gelE-sprE* virulence locus. The number of predicted OG1RF genes (2384) in common with V583 was significantly lower compared to 2474 genes identified in a previous report [23]. This instigated us to perform a more detailed analysis to identify the cause of this discrepancy. For this purpose we performed BLASTN comparison to V583 of 2558 genes (Table S2) predicted using EasyGene 1.2 [32], which showed an overall identity (∼96.5%) between the CGH and the BLASTN analysis. An interactive Genewiz map [33] of OG1RF CGH and BLASTN (Genbank ABPI00000000) analysis compared with V583 is accessible at; http://ws.cbs.dtu.dk/cgi-bin/gwBrowser-0.91/edit.cgi?hexkey=6561d07e713b77fe75aa3403798e36c1. Moreover, BLASTN comparison of the annotated genes of V583 with the OG1RF genome sequence using 75% sequence identity across an entire CDS (Table S3) identified 2385 orthologous genes, confirming the results obtained by CGH and EasyGene 1.2 analysis.

### Transcriptional analysis

A rich laboratory medium (2xYT) was used as the reference culture medium since it is considered to contain a minimum of infection relevant biological cues [17]. The growth capacity in urine was compared to that in the 2xYT medium by CFU counts (Figure S1). We found that growth in urine was slightly slower, and the cell density obtained was about one log lower than in 2xYT. For the transcriptional analysis, the three strains were grown in 2xYT to a cell density ∼1×10^7^ before exposure to either pre-warmed urine or 2xYT (control). Samples were collected after 5 (*t_5_*) and 30 (*t_30_*) minutes growth. The obtained log_2_-ratios and *q*-values for the three strains during growth in urine compared to 2xYT are listed in Table S1.

### Growth in urine vs. 2xYT triggers global transcriptional changes for both pathogenic and probiotic *E. faecalis*

**Figure 4:**
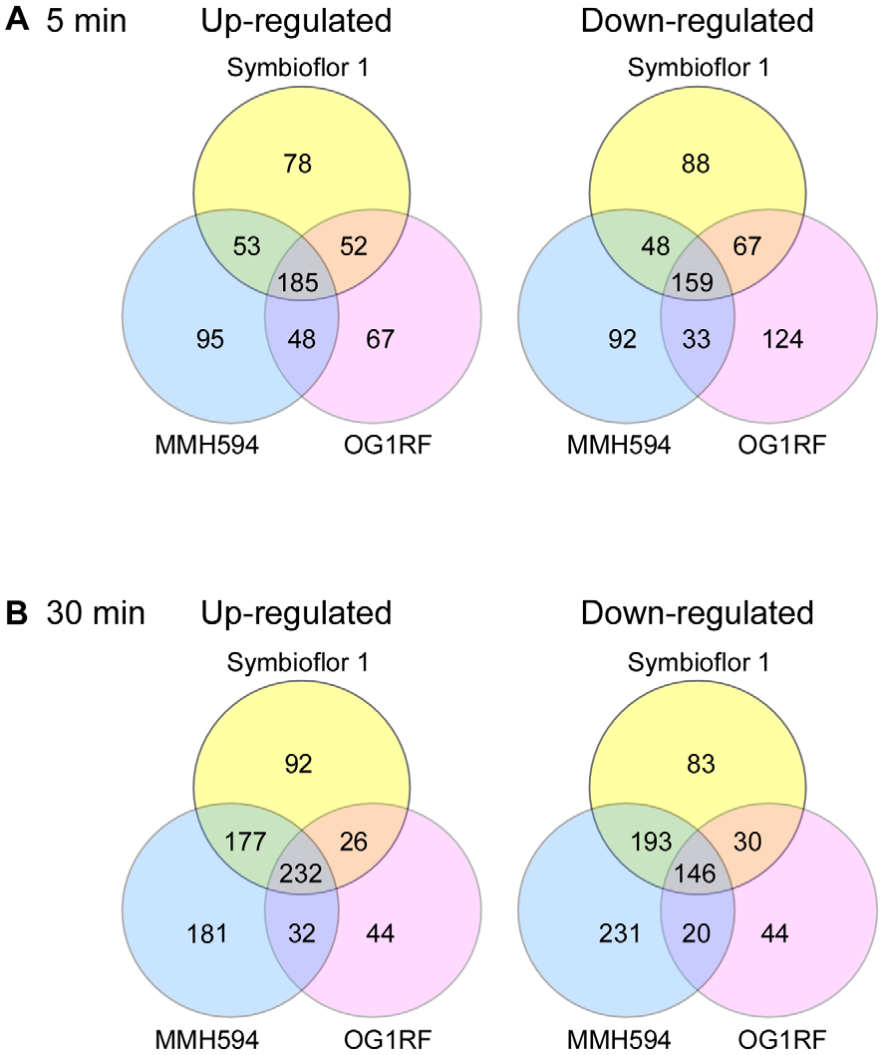
Distribution of differentially expressed genes during growth in urine. Venn diagram showing the number of unique and common up- and down-regulated genes in MMH594, OG1RF and Symbioflor 1 when grown in urine compared to 2xYT after A: 5 minutes (*t_5_*) and B: 30 minutes (*t_30_*).

**Figure 5:**
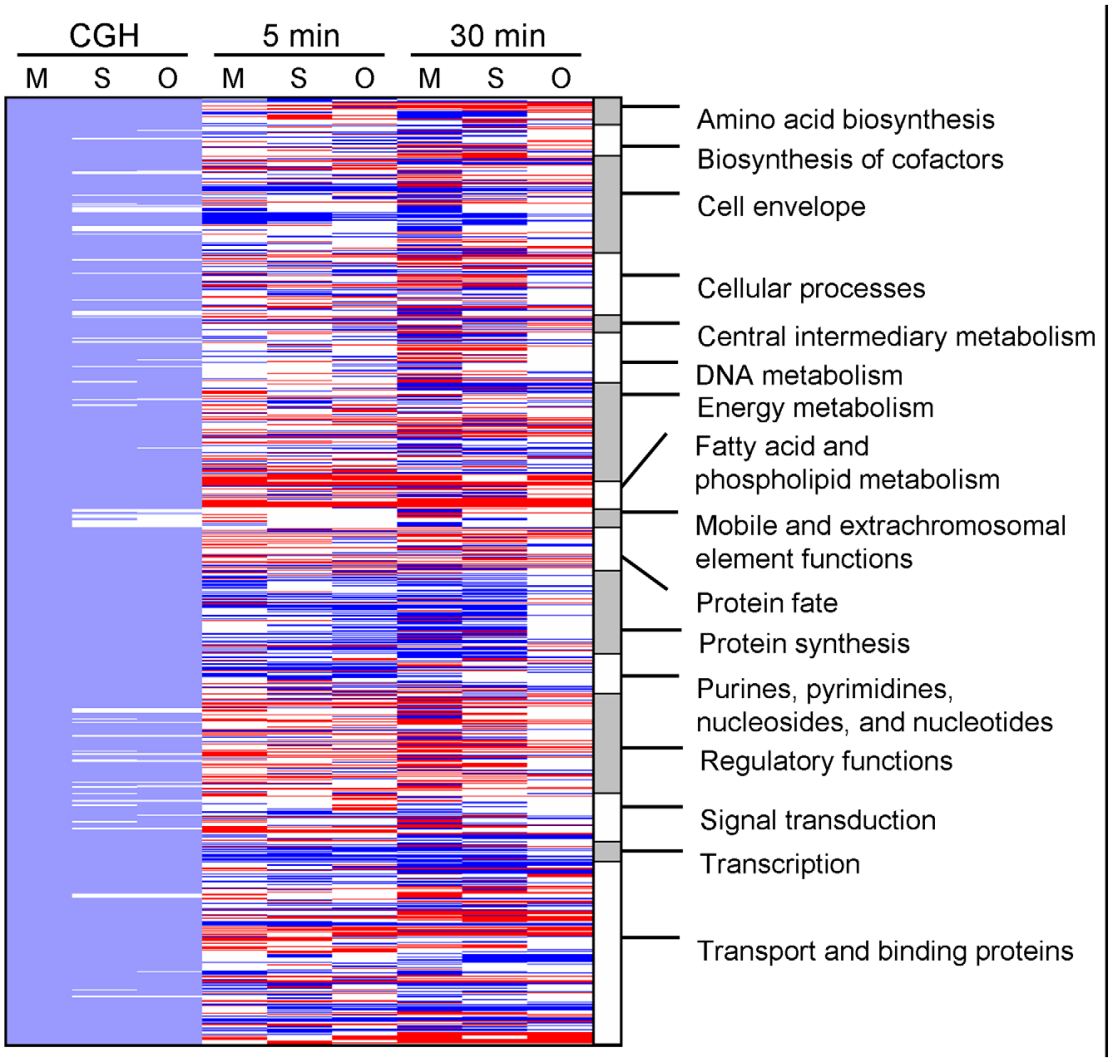
Heat map of CGH data and differentially expressed genes during growth in urine. Heat map visualizing the regulated genes in MMH594 (M), Symbioflor 1 (S) and OG1RF (O) when grown in urine compared to in 2xYT. The comparative genome hybridization (CGH) results for the respective regulated genes are shown in columns 1–3 (light blue: present gene, white: divergent gene). Genes found to be significantly regulated are indicated by either red (up-regulated), or blue (down-regulated). Genes regulated after growth for 5 minutes (*t_5_*) in urine compared to in 2xYT are listed in columns 4–6 and after 30 minutes (*t_30_*) in columns 7–9. The functional categories are sorted alphabetically (column 10). Significantly regulated hypothetical genes and genes encoding proteins with unknown function are not included in this heat map.

The microarray results revealed changed expression in most functional gene categories for all three strains. At *t_5_*, 713 genes were differentially expressed in MMH594, 735 in OG1RF and 730 in Symbioflor 1. 344 of these regulated genes were common for all three strains (Figure 4A). At *t_30_*, the number of regulated genes increased dramatically to 1212 genes in MMH594 and 979 in Symbioflor 1. However, in OG1RF the number of regulated genes decreased to 574 after 30 minutes growth in urine. It is possible that the reduced number of regulated genes in OG1RF at *t_30_* reflects a more rapid adjustment to the new growth environment, which potentially can be advantageous for the establishment of an infection. This notion was further supported by the swift derepression of macromolecular biosynthesis (*e.g.* protein synthesis) in OG1RF, compared to the two other strains. A total of 378 differentially expressed genes were common for MMH594, OG1RF and Symbioflor 1 at *t_30_* (Figure 4B). Of the 596 genes that appeared unique to MMH594, 153 were differentially expressed at one or both time points during growth in urine. None of the genes unique to OG1RF or Symbioflor 1 were differentially expressed. The heat map in Figure 5 presents an overview of the regulated genes within each functional category for the three strains. An overview of the number of regulated genes within each functional category is given in Figure S2.

### Transcription of metabolic pathways during growth in urine

Prior to the current study, no comprehensive investigation regarding which substrates or metabolic processes that confer growth of *E. faecalis* in urine existed. The transcriptome data (Table S1) was thus examined to identify metabolic pathways that showed specific responses during growth in urine.

With respect to carbon metabolism the genes encoding the main glucose uptake-system, mannose phosphoenolpyruvate phosphotransferase (PTS) *mptBACD* (EF0019-22) [34] were down-regulated in all three strains at *t_30_*. This is consistent with a recent metabolomic investigation which showed that urine from healthy adults contains glucose concentrations in the range of 0.2–0.6 mM [35]. Such concentrations of glucose is below the threshold for release of carbon catabolite repression (CCR), and the cells thus initiate use of less preferred carbon and energy sources [36]. This implied that substrates besides glucose might play a role for growth of *E. faecalis* in urine. However, of the loci known to be subject to catabolite control protein A (CcpA) mediated CCR, only the genes encoding citrate metabolism (EF3322-15) [37] were positively modulated in MMH594 and OG1RF at both time points and at *t_5_* in Symbioflor1. At *t_30_* EF3322-15 only showed a slightly (not statistically significant) enhanced expression in Symbioflor 1. The content of citrate in human urine is in the range of 1–2 mM [38], which suggests that citrate metabolism is important for *E. faecalis* during growth in urine.

PTS systems facilitate uptake of diverse sugars in *E. faecalis*. Two operons encoding a sucrose uptake PTS-system (EF1602-01) and sucrose metabolism (EF1603-04) showed consistent up-regulation in all three strains. Dietary sucrose is normally degraded in the intestinal lumen and absorbed as glucose and fructose, but a previous study has shown that even healthy individuals have µM sucrose content in their urine [39]. Moreover, the sugar content in urine increases with high sugar diet. Once sucrose is present in the bloodstream it is not metabolized further, but removed from the blood via the renal capillaries and excreted into the urine, reaching concentrations of 70 to 200 µM [39]. Interestingly, EF1603-04 knock-out mutants show reduced virulence in a *Caenorhabditis elegans* infection model [40], [41]. All three strains showed elevated expression of the major facilitator family transporter (EF0082) proposed to function in import of phosphorylated sugars [42] and glycerol [43], which implies that such substrates might contribute to growth in urine.

Transcriptome analysis conducted on an *E. coli* asymptomatic bacteriuria strain revealed an important role of amino sugar and amino acids present in urine as growth substrates [44]. The transcription of *nagB* (EF0466) and *nagA-1* (EF1317) involved in N-acetyl glucosamine metabolism was elevated, implying that these substrates were utilized by *E. faecalis* during growth in urine. A massive down-regulation of *glmS* (EF2151), which is responsible for conversion of fructose-6P into glucosamine-6P using glutamine as a nitrogen source, could signify glutamine constraints.

Growth in urine also had an impact on pyruvate metabolic pathways and certain changes were strain specific. For OG1RF and Symbioflor 1, we observed increased expression of L-lactate dehydrogenase (*ldh-*1; EF0255), whereas expression of *adhE* (EF0900), involved in ethanol formation was reduced. The *pflAB* (EF1612 and EF1613) genes responsible for formate formation were reduced in MMH594 and Symbioflor 1 at *t_30_*. In all strains the *lutABC* operon (EF1108-1110), involved in metabolism of L-lactate like substrates was up-regulated. The pyruvate dehydrogenase complex gene-cluster *pdhAB, aceF* and *lpdA* (EF1353-56) involved in acetyl-CoA biosynthesis showed consistent up-regulation in all three strains at *t_30_*. Moreover, the *ackA* gene (EF1983) responsible for conversion of acetyl-phosphate to acetate and ATP was significantly down-regulated, perhaps as a consequence of increased acetate production due to elevated activity of the citrate metabolism (EF3322-15) [37]. It is thus conceivable that the increased acetyl-CoA formation serves to supply either the FASII biosynthesis, or the citrate metabolism.

### Transport and biosynthesis

Compared to the rich 2xYT medium the growth rates were significantly lower in urine, and moreover, the growth halted one order of magnitude below that in 2xYT (Figure S1). For *E. coli* it has been demonstrated that growth in urine is restricted by availability of one specific cofactor, namely iron [44]. We were thus interested to see whether the transcriptional responses with respect to transport and biosynthesis processes in *E. faecalis*, could reveal candidate nutrients or co-factors whose availability restrict growth of *E. faecalis* in urine.

Human urine contains significant amounts of creatine, creatinine, and glycine, while other amino acids like histidine, glutamine, methionine, proline, glutamate, arginine and branched chain amino acids (bcaa) are present at lower concentrations [45]. The CGH-results indicate that MMH594 and Symbioflor 1 have similar requirements for amino acids as OG1RF, which was shown to be auxotrophic for amino acids like histidine, isoleucine, methionine, and tryptophan [24]. Also, some *E. faecalis* strains require arginine, glutamate, glycine, leucine, or valine [24], and are capable of utilizing certain amino acids as energy and carbon source [46], [47]. However, transcription of the genes encoding catabolism of arginine (EF0104-7 and EF0108) and serine (EF0097-100) was significantly reduced at *t_30_*, indicating a shift towards protein synthesis rather than energy metabolism.

According to our data, the transcription of several genes encoding oligo-peptide ABC-transporters (EF0907, EF0909-12 and EF3110-06) was enhanced at *t_30_*, while the transcription of three amino acid permease genes (EF0635, EF0929 and EF2377) and two operons encoding amino acid transporters (EF0247-46 and EF0761-60) was reduced in all three strains at the same time point (Table S1). These observations indicate that *E. faecalis* meets its demand for certain amino acids by acquiring oligo-peptides during growth in urine. However, the *gln*-operon encoding glutamine/glutamate transport system (EF1120-17) [48] was up-regulated in all strains at both time points, suggesting that glutamate/glutamine from urine were utilized. This was further supported by the observed reduced expression of the glutamine synthase operon *glnRA* (EF2160-59) in all three strains and glutamate synthase *gltA* (EF2560) in OG1RF and Symbioflor 1 at *t_30_*. On the contrary, the increased expression of *cysK* (EF1584) implies that cysteine is scarce in urine, which also is in accordance to the metabolomic analysis of human urine [45].

An operon comprising a putative amino acid ABC transporter (EF0893-92) and a putative aspartate aminotransferase (EF0891) was highly up-regulated in all strains. The latter gene is predicted to facilitate the conversion of aspartate and alpha-ketoglutarate to oxaloacetate and glutamate, which may also in turn explain the down-regulation of the above mentioned *gltA*. Furthermore, the transcription of a gene encoding methionine synthase (EF0395) was enhanced in all three strains. These results are consistent with the metabolomic analysis of human urine which showed that aspartate is 5-fold more abundant than methionine [45]. These observations imply that aspartate might serve a key role for nitrogen metabolism of *E. faecalis* in urine. Thus, it appears that *E. faecalis* scavenge available peptides and amino acids, which in turn are sequentially hydrolyzed and transaminated in order to fuel the pool of depleted amino acids.

Urinary tract pathogenic bacteria like *E. coli* (UPEC), have pathogenic islands dedicated to acquisition of limited nutrients and biometals [49]. Manganese is one such factor which is essential for the fermentative metabolism of lactic acid bacteria (LAB) [50], [51]. The up-regulation of the main manganese scavenging mechanism encoded by *efaCBA* (EF2074-76), accompanied by two other genes (EF1057 and EF1901) encoding Mn^2+^/Fe^2+^ transporters in all strains at both time points is a clear indication that *E. faecalis* scavenged manganese. The content of manganese in human urine is in the nano molar range [52], while the optimal concentration for *E. faecalis* is in the micro molar range [53]. Thus manganese may be restrictive for the growth of *E. faecalis*. This in turn can affect the virulence of the bacterium and *efaCBA* has indeed been shown to be implicated in virulence [54]. Notably, for MMH594 a potential auxiliary Mn-uptake system (EF 0575-78) [55], located within the PAI also showed highly elevated expression, indicating that PAI harboring strains might be better equipped to cope with manganese depleted environments.

In addition to the above mentioned Mn^2+^/Fe^2+^ transporters, our experiments also revealed an enhanced expression of several other genes involved in iron transport; the *feoAB* (EF0475-76) and *ceuBCD* and *fatB* (EF3085-82) operons were up-regulated in all strains at *t_30_*. Another gene involved in iron transport, *feuA* (EF0188) was down-regulated in all strains at *t_5_*, but was up-regulated in MMH594 and OG1RF at *t_30_*. Interestingly, a third iron transport encoding operon (EF0191-93) was down-regulated in all three strains at *t_5_*, while up-regulated at *t_30_* in OG1RF only. Iron is one of the main limiting factors for *E. coli* growth in urine and the addition of iron to urine increased the maximum growth extensively [18], [19]. LAB, on the other hand comprise one of the very few groups of bacteria for which iron is not an essential growth factor [51]. Even so, our data suggest a potentially important role of iron acquisition and metabolism during growth in urine.

### Stress response of *E. faecalis* towards exposure to urine

Proteomic analyses with systematic exposure to various stresses have previously identified six genes encoding general stress response proteins (GSPs) which were up-regulated in *E. faecalis* by a wide variety of environmental stimuli [56]. The enhanced expression of all the GSP-encoding genes at one or both time points in the present study indicates that the bacterium experienced a multitude of stress factors upon the encounter with urine. This impression was further substantiated by the significantly differential transcription of a large number of genes with a proven or predicted function in other stress responses in *E. faecalis* [112]–[120] (Table S1 and S4).

The gene encoding Gsp62 (EF0770; hypothetical protein) was the only GSP which showed a significantly enhanced expression in all strain at both time points. The stress- and starvation inducible *gls24* operon (EF0076-81) was significantly up-regulated at both time points in OG1RF and Symbioflor 1, while partly up-regulated in MMH594. Inactivation of *gls24* and *glsB* (EF0079 and -80, respectively) has been reported to have a pleiotrophic effect on cell morphology and stress tolerance in *E. faecalis* [57]. A *gls24* disruption mutant has also been shown to be highly attenuated in animal infection models [58], [59]. MMH594 contains two additional *gls24*-like genes within the PAI (EF0604 and PAIef0055). Both gene were up-regulated at *t_30_* and might possibly contribute to the fitness of MHH594 during growth in urine.

An organic hydroperoxide resistance protein, *ohr* (Gsp65; EF0453) was up-regulated in MMH594 at *t_5_*, and in all three strains at *t_30_*. An *ohr* mutant has previously been shown to be less resistant to the oxidative stress generated by 20 mM Tertiary-Butylhydroperoxide, suggesting that Ohr may be implicated in oxidative stress resistance in *E. faecalis* [60]. Interestingly, the microarray data revealed differentially expression of an arsenal of genes holding putative roles in oxidative stress response in *E. faecalis* (Table S1 and S4). The enhanced transcription of genes involved in oxidative stress response during exposure to urine is interesting. Especially in light of an observed adaptation to lethal challenges of H_2_O_2_ by pretreatment with sublethal concentrations of H_2_O_2_ [61], and a reported link between oxidative stress response and survival within macrophages in enterococci [62], [63], [64]. Furthermore, it has been demonstrated that purified lipoteichoic acids from *E. faecalis* induced proliferation and production of nitrous oxides and cytokines by a subpopulation of basal urothelial cells [65], [66]. It is thus tempting to speculate that urine act as a cue to trigger oxidative stress-protection by *E. faecalis*, in order to render increased resistance against certain host defense mechanisms in the urinary tract.

### Modifications to the cell envelope caused by growth in urine

When infecting a host, the integrity and composition of the cell envelope of the bacterium are important to avoid damage by the host defense systems [67], [68]. In the case of *E. faecalis*, it has been demonstrated that important processes in the interaction with the host *e.g.* recognition by immune system mechanisms and innate immune evasion, involve specific cell envelope structures like lipotechioc acids [69], and cell wall and capsular polysaccharide determinants [70], [71].

During growth in urine, signs of adaptation to this new growth environment were evident for several genes important for the cell membrane composition and surface related structures (Table S1). We observed an immediate response to urine by the up-regulation of two gene clusters (EF0282-84 and EF2886-75) responsible for type II fatty acid biosynthesis (FASII) and isomerization of membrane phospholipids. Most of these genes were up-regulated in all strains at *t_5_* and *t_30_*. Interestingly, these gene clusters have previously been shown to be up-regulated in response to growth in blood [72] and to exposure to the cell membrane detergent SDS [73]. Furthermore, the FASII genes were down-regulated in response to exposure to NaCl (Solheim, unpublished data), bovine bile, and SDS and bovine bile in combination [73], indicating that several different external stressors triggers remodeling of the fatty acid composition in the cell membrane.

In addition to the FASII pathway, a regulation of three genes encoding lipases (EF0169, EF1683 and EF3191) and two genes encoding cardiolipin synthetases (EF0631 and EF1608) further indicates both degradation and processing of fatty acids (Table S1). It is possible that the lipolytic activity is connected to a modulation of the FASII genes, as it recently was demonstrated that *E. faecalis* can utilize available fatty acids from the environment in their membrane biogenesis [74]. However, there are only trace amounts of free fatty acids in urine [38], and it is therefore more likely that the remodeling of the fatty acid composition in the cell membrane is a more general stress response in *E. faecalis*, while the lipases may play a more specialized role in virulence. A recent study by Walecka and co-workers revealed that a higher percentage of invasive *E. faecalis* isolates produce lipases compared to non-invasive isolates [75], indicating a central role for lipase activity during invasive infection. Notably, Symbioflor 1 showed a more enhanced expression of genes encoding lipases compared to the pathogenic strains.

The ability of *E. faecalis* to adhere and develop biofilm is thought to be important for its potential to cause UTI and other infections [76]. In our experimental design, the cells were cultivated planktonically. We were thus interested in assessing whether genes implicated in adherence or biofilm formation would be modulated by human urine. The gene encoding the maltose PTS system *malT* (EF0958) and the cognate operon *bopABCD*/*malPBMR* (EF0957-54), are involved in biofilm formation [77], [78], and were partly up-regulated in OG1RF at *t_5_*. Another gene important for biofilm production and the initial attachment stage for binding to abiotic surfaces is a sortase A encoding gene, *srtA* (EF3056) [79], [80]. This gene showed an enhanced expression in MMH594 at *t_30_* and Symbioflor 1 at both time points. Interestingly, an *srtA* mutant showed a slightly attenuated virulence during UTI in mice [81]. However, among the genes encoding potential substrate proteins of SrtA, only EF2713 was up-regulated at *t_5_* in MMH594, whereas EF3314 showed an enhanced expression in Symbioflor 1 at both *t_5_* and *t_30_*. This latter gene encodes a protein recently shown to be important for the pathogenicity of *E. faecalis* [82], and it is noteworthy that the only strain which showed an enhanced expression of this gene, was the probiotic strain.

Mohamed *et al.* [83] demonstrated that a knockout mutant of the secreted antigen *salB* (EF0394) in OG1RF showed reduced biofilm formation in BHI, but enhanced biofilm production in the presence of serum or fibronectin. The authors also showed that the *salB* mutant was able to bind to the extra cellular matrix (ECM) proteins collagen type I and fibronectin, whereas wild type OG1RF did not bind these ECM proteins [83]. Furthermore, they showed that a *salA* (EF3060; secreted lipase) mutant also produced slightly less biofilm than wild type OG1RF, while binding to ECM was unaffected. During growth in urine *salA* was down-regulated in all strains at both time points, while *salB* was down-regulated in all strains at *t_5_*, and in MMH594 at *t_30_*. Mohamed and co-workers [83] speculated that under certain conditions a down-regulation of *salB* would be sufficient to see similar effects as was seen for the *salB* mutant, thus it is possible that the expression of *salB* and possibly also *salA* is reduced in response to urine in order to promote colonization of the urinary tract.

At *t_5_*, a gene encoding the major autolysin of *E. faecalis*, *atlA* (EF0799) was down-regulated in all three strains. An *atlA* deletion mutant of OG1RF showed delayed biofilm formation, reduced attachment on plastic surfaces and longer chains than the wild type OG1RF [79], [84]. AtlA is also essential for DNA release and biofilm accumulation, which is needed for the development of a mature biofilm in *E. faecalis* [79], [85]. MMH594 and Symbioflor 1 contain a second peptidoglycan hydrolase encoding gene *atlB* (EF0355), which have been shown to compensate for the absence of AtlA in autolysis and cell separation [84]. *atlB* was down-regulated at *t_30_* in MMH594, while not differentially expressed in Symbioflor 1. The lowered expression of *atlA* and *atlB* may also be connected to reduced cell wall synthesis indicated by down regulation of several genes responsible for peptidoglycan biosynthesis (Table S1), which again is consistent with the significantly lower growth rate in urine compared to 2xYT.

Bacterial surface proteins are key players in host-pathogen interactions [81]. Therefore, the change of membrane bound proteins might alter the bacterium's potential of causing an infection. Regulation of several genes encoding proteins bound to the cell membrane or cell surface *i.e.* membrane proteins and lipoproteins was observed for all three strains (Table S1). Moreover, most microbial surface components recognizing adhesive matrix molecules (MSCRAMMs) and cell-wall anchor family proteins [86] including the endocarditis- and biofilm-associated pilus (*ebp*) [87], [88] were either down-regulated, or not differential regulated (Table S1).

A gene encoding a chitin binding protein (EF0362) and one encoding a chitinase (EF0361) were up-regulated in all three strains at *t_5_*. The direct function for these genes in response to urine is not clear, however a homologous protein GbpA in *Vibrio cholerae* was shown to facilitate binding to the chitin monomer N-acetylglucosamine [89], a sugar residue found on the surface of epithelial cells [90], [91], [92], which line the cavities and surfaces of structures including the urinary tract. Hence, it is possible that biological cues in urine trigger the up-regulation of these genes as an initial step of adherence to uroepithelial cells. Interestingly, growth of *E. faecalis* V583 in blood triggered an even more enhanced transcription of these two genes [72], but a functional study of these genes would be required to elucidate any function related to enterococcal virulence.

Most of the genes within a cluster responsible for the production of a serotype-determining exopolysaccharide (EF2198-2177; *epa*) [93], [94] were down-regulated both at *t_5_* and *t_30_* in the three strains. An OG1RF Δ*epaB* mutant has previously been reported to show reduced virulence in mice [95], higher susceptibility to phagocytic killing [71], and decreased biofilm formation compared to the wild type [71], [96]. Furthermore, Singh *et al.* recently showed that the *epaB* mutant was less competitive compared to the wild type in a model of UTI in mouse [97]. However, it is possible that the exopolysaccharide production is body-site dependent, and could be more pronounced in *E. faecalis* that have reached the glomerular basement membrane in kidneys, which is a preferred site for *E. faecalis* colonization [97], [98].

The serotype 2 capsular polysaccharide (*cps*) [99], which constitutes an important virulence factor that enables *E. faecalis* to evade phagocytic killing, by masking the lipoteichoic acids [70], is absent in both OG1RF and Symbioflor 1 (Figure 3 and Table S1). Intriguingly, the *cps* gene cluster (EF2495-85) was down-regulated in MMH594 at *t_30_*, which is similar to the response observed in V583 growing in blood [72]. It is tempting to speculate that a basal capsular polysaccharide production could be sufficient to protect *E. faecalis* from complement-mediated opsonophagocytosis, especially in infected tissues where micro-colonies or biofilm develop.

In sum, the human urine milieu appears to instigate a drastically altered composition of the cell envelope and cell surface structures, some of which might be advantageous or required for establishment of *E. faecalis* UTI.

### Virulence traits and Regulatory genes

A number of genetic traits have been identified to contribute to virulence in *E. faecalis* [5], [6], [8], [25], [59], [71], [99], [100], [101], [121], [122]. The expression of selected virulence genes in MMH594 during growth in urine have previously been examined by real-time quantitative PCR (QPCR) [17]. More recently, a new QPCR study of the expression in several strains including MMH594 during growth in urine was published [102]. The two studies show some differences in gene expression in MMH594, *e.g.* of a gene encoding the enterococcal surface protein Esp (PAIef0056). Shepard and Gilmore [17] found an enhanced expression of *esp*, while Carlos *et al.* [102] found a reduced expression of the same gene. In the present study, we found that the *esp* gene was not significantly differentially expressed. Indeed, QPCR appear to be more sensitive and have a broader detection range than microarray, but the deviating results still seem to imply a problem when comparing these types of experiments. Shepard and Gilmore [17] reported a growth-phase dependent difference in the expression of the virulence genes tested. Hence, the differences observed between these three similar experiments are most likely due to the different methods used for cultivation. Our aim was to investigate the immediate effect on actively growing *E. faecalis* cells upon the first encounter of urine. We revealed a significant impact on the transcription of a number of virulence related traits connected to stress, co-factor acquisition, and cell surface structures (described above), and a summary of these genes can be found in Table S5.

The *fsr* quorum sensing system has been shown to coordinate expression of the virulence factors *gelE* (encoding a gelatinase) and *sprE* (encoding a serine protease) during infection of *C. elegans* and in mouse peritonitis models [25], [101], and several other genes were differentially expressed in wild type OG1RF compared to an *fsrB* mutant, indicating a more complex regulatory network [103]. Consistent with previous observations [17], we detected a modest up-regulation of the *fsrABC* genes (EF1822-20) in MMH594 at *t_30_*. The *fsrA* gene was also up-regulated at *t_5_*. In addition, the downstream *gelE* (EF1818) was down-regulated at *t_5_* in MMH594. No regulation of these genes was observed in OG1RF (the genes are divergent in Symbioflor 1). However, due to the fact that several of the *fsr*-genes had been excluded from the data analysis as a result of the number of functional spots in the latter strain (see Materials and methods), the expression of *fsrB* was verified by real time quantitative PCR (QPCR; Figure S3). The QPCR analysis of *fsrB* revealed that the log_2_-ratio was below the threshold for significant differential expression in MMH594. Differential expression of *fsrB* was however, observed in OG1RF. These results are in line with previous findings which suggest that growth in urine promotes transcription of the *fsr*-quorum sensing system [17]. Quorum sensing regulatory cascades are characteristically initiated by elevated expression of a regulatory unit, in this case the *fsr*-operon, of which the most likely consequence would be the subsequent induction of the *fsr*-regulon.

The PAI is significantly more prevalent among infection-derived isolates compared to *E. faecalis* from other sources [22], [28], [30], [104]. Moreover, the contribution of PAI-related genes to the pathogenicity of *E. faecalis* has been experimentally determined for certain traits, such as *araC*, cytolysin, *esp* [5], [100], [105]. In the genome of the three strains used in this study only MMH594 contains the entire enterococcal PAI (129 genes, of which 125 were represented on the array). Fifteen PAI genes were down-regulated, while twenty PAI genes including manganese transporter (EF0575-77), *gls24* (EF0604) and a bile salt hydrolase (BSH; EF0521) were up-regulated in MMH594 at *t_30_*. The latter gene was also the only PAI gene which showed an enhanced expression in Symbioflor 1. The BSH and the Gls24 starvation-inducible protein are factors that have been hypothesized to be advantageous in colonization of the gastrointestinal tract, and our results demonstrate that potential virulence-, stress- and fitness-genes located in the PAI do in fact respond to an infection-relevant milieu like urine. However, the exact function of these genes in the pathogenicity of *E. faecalis* remains to be elucidated. Moreover, transcripts were detected for a substantial number of PAI genes, implying that their mere presence and basal expression might also be important during UTI.

In conclusion, a significant proportion of the transcriptional responses seen during growth in urine were common for the three different strains examined, and the main differential regulation was observed among genes related to stress responses, energy metabolism, acquisition of trace metals, and a drastic modification of the cell envelope. Despite the failure to identify pathogen-specific *E. faecalis* genes, the overall similarity between the transcriptional responses of pathogenic and non-pathogenic strains presented here, implies that the pathogenic potential of an *E. faecalis* strain may in fact be determined by presence or absence of specific genes, rather than the level of expression of such traits.

## Materials and Methods

### Bacterial strains and growth conditions

Bacterial strains used in this study are listed in Table 1. The growth capacity of six *Enterococcus faecalis* strains was examined. Three of these strains were selected for transcriptional profiling based on their origin. For all experiments *E. faecalis* strains were streaked on a 2xYT agar plate (1% (w/v) yeast extract, 1.6% (w/v) tryptone and 1% (w/v) NaCl) and incubated at 37°C over night (ON). Four individual colonies were then inoculated into the same tube of 5 ml 2xYT medium and grown ON without shaking at 37°C. For growth in urine, human urine was collected from four healthy men and women who had no history of UTI or antibiotic use in the last 6 months. The urine was pooled with equal amounts from each volunteer, centrifuged at 12000×g and sterilized twice by filtration (0.22 µm-pore size). Since the composition of human urine may potentially be variable, samples were collected on three separate days for three replicate experiments and used within the next day.

**Table 1:** Bacterial strains used in this study.

Strain	Country	Source	Isolation site	MLST		Characteristics	Reference
				CC	ST		
Baby isolate 62	Norway	Non-hospitalized person <1 year	Feces	S	66	Tet^R^	[106]
MMH594	USA	Hospitalized patient	Blood	6	6	Ery^R^,Gen^R^, hospital outbreak	[21]
OG1RF	USA	Laboratory strain		21	1	Rif^R^, Fus^R^	[24]
Symbioflor 1	Germany	Non-hospitalized person	Feces	25	248	Probiotic	[20]
V583	USA	Hospitalized patient	Blood	6	6	Ery^R^, Gen^R^, Van^R^	[123]
179Vet	Norway	Animal_dog	Urine	9	9	Multi-resistant*	[29]

CC =  clonal complex; Ery =  erythromycin; Fus =  fusidic acid; Gen =  gentamicin; MLST =  multilocus sequence typing; R =  resistance; Rif =  rifampicin; S =  singleton; ST =  sequence type; Tet =  tetracycline; Van =  vancomycin.

*Tested against 16 different antibiotics, of which it was susceptible only to ampicillin.

### Growth measurement

The six *E. faecalis* strains were pre-cultured as described above. ON cultures were diluted 1000× in either preheated urine (37°C) or in preheated 2xYT medium and incubated ON. These cultures were then diluted 1000× in either preheated urine or 2xYT, and cell growth was measured spectrophotometrically with a Bioscreen instrument (Bioscreen C) and by plating and colony forming units (cfu) counts. Growth experiments measured spectrophotometrically were performed in triplicates with a total volume of 300 µl of bacterial inoculum in fresh urine or 2xYT medium. Wells containing sterile urine/2xYT were used as negative controls. Cultures were incubated at 37°C and optical density 600 nm (OD_600_) was measured at 15-min intervals for 24 hours. To determine CFU/ml, viable cell counts were performed as follows: ON cultures were inoculated (1000× dilution) in preheated urine. Samples were collected immediately after inoculation, and after 2, 4, 6, 8, 10 and 24 hours for 2xYT, and also after 15 hours for urine. The number of CFU/ml was estimated by averaging the colony count values in two replicates per strain after ON incubation at 37°C.

### Cultivation and sampling prior to microarray analysis

The three selected *E. faecalis* strains, MMH594, OG1RF and Symbioflor 1 were pre-cultured as described above. The cultures were then diluted 1000× in 250 ml pre-warmed 2xYT medium and incubated further at 37°C. When the culture reached OD_600_ = 0.1 the cultures from each strain was split in two and centrifuged (10000×*g* for 3 min at 37°C). For the control cultures the pellets were resuspended in 100 ml pre-warmed 2xYT (37°C) whereas for the test culture the pellet was resuspended in 100 ml pre-warmed urine (37°C). Samples (45 ml) of each culture were collected immediately after the resuspension in urine (t_5_), and after 30 min (t_30_) by centrifugation (8000×*g* for 2 min at 37°C), and the pellets were immediately frozen in liquid nitrogen and kept at −80°C prior to RNA extraction.

### RNA isolation, cDNA synthesis, fluorescent labeling and hybridization

Total RNA was isolated by FastPrep (Bio 101/Savant) and RNeasy Mini kit (QIAGEN) as previously described [72]. The concentrations of the RNA samples were measured by using the NanoDrop (NanoDrop Technologies), and the quality was assessed by using the RNA 600 Nano LabChip kit and the Bioanalyzer 2100 (Agilent Technologies). cDNA was synthesized and labeled with the Fairplay III Microarray labeling kit (Stratagene) according to the manufacturer's protocol, with the following modifications: For each labeling reaction, 10 µg of total RNA and 500 ng of random primers were initially preheated at 70°C for 10 min. A reverse transcription-PCR mixture (10× AffinityScript RT buffer, a 20× deoxynucleoside triphosphate mixture, 0.1 M dithiothreitol, 20 U RNase block, and AffinityScript HC RT) was added to the annealed primers and RNA, and the reaction mixture was further incubated for 3 h at 42°C. After labeling, 1 µL of hydroxylamine (Sigma Aldrich) was added to quench the coupling reaction, and the reaction mixture was incubated 10 min. at room temperature. 70 µL RNase-free water was then added, and unincorporated dyes were removed from the samples by using the QIAquick PCR purification kit (QIAGEN). Labeled samples were then dried, prior to resuspension in 140 µl hybridization solution (5× SSC, 0.1% (w/v) SDS, 1.0% (w/v) bovine serum albumin, 50% (v/v) formamide and 0.01% (w/v) single-stranded salmon sperm DNA) and hybridized for 16 h at 42°C to the array in a Tecan HS 400 pro hybridization station (Tecan). Arrays were washed twice at 42°C with 2× SSC +0.2% SDS, and twice at 23°C with 2× SSC, followed by more stringent washes at 23°C with 0.2× SSC and with filtrated H_2_O. Three replicate hybridizations were performed with three separate batches of RNA. The three batches of RNA were obtained in three separate growth experiments. The Cy3 and Cy5 dyes (Amersham) used during cDNA synthesis were swapped in two of the three replicate hybridizations. All samples in the three experiments were co-hybridized with control samples collected at equal time points (*e.g. t_5_* was hybridized along with *t_5_*). Hybridized arrays were scanned at wavelengths of 532 nm (Cy3) and 635 nm (Cy5) with a Tecan scanner LS (Tecan). Fluorescent intensities and spot morphologies were analyzed using GenePix Pro 6.0 (Molecular Devices), and spots were excluded based on slide or morphology abnormalities.

### Microarrays

The microarray used in this work has been described previously [106]. The microarray designs have been deposited in the ArrayExpress database with the accession numbers A-MEXP-1688 and A-MEXP-1765.

### Data analysis

Downstream analysis was done by the LIMMA package (www.bioconductor.org) in the R computing environment (www.r-project.org). Preprocessing and normalization followed a standard procedure using methods described by Smyth & Speed [107]. Testing for differential expressed gene was done by using a linear mixed model as described in Smyth [108]. A mixed-model approach was chosen to adequately describe between-array variation and still utilize probe-replicates (3 replicates of each probe in each array). An empirical Bayes smoothing of gene-wise variances was conducted according to Smyth *et al* [109]. For each gene, the p-value was adjusted to control the false discovery rate; hence, all p-values displayed are FDR-adjusted (often referred to as *q*-values). A gene was found to be significantly regulated if *q*<0.01 and the log_2_-ratio was similar to or above 0.5, or similar to or below −0.5. Genes represented with less than 1 spot on one or more arrays were excluded from the final results (NA).

### Comparative genomic hybridization

Genomic DNA was isolated by using the FP120 FastPrep bead-beater (BIO101/Savent) and the QiaPrep MiniPrep kit (Qiagen), as previously described [106], and then labeled and purified with the BioPrime Array CGH Genomic labeling System (Invitrogen) and Cyanine Smart Pack dUTP (PerkinElmer Life Sciences), according to the manufacturer's protocol. Standard methods in the LIMMA package [107] in R (http://www.r-project.org/), available from the Bioconductor (http://www.bioconductor.org) were employed for preprocessing and normalization. Within-array normalization was first conducted by subtracting the median from the log-ratios for each array. A standard loess-normalization was then performed, where smoothing was based only on spots with abs(log-ratio) <2.0 to avoid biases due to extreme skewness in the log-ratio distribution. For the determination of present and divergent genes a method that predicts sequence identity based on array signals was used, as described by Snipen *et al*. [110]. A threshold of 0.75 was used in order to obtain a categorical response of presence or divergence, *i.e.* genes with *Sb*-value >0.75 were classified as present, while genes with *Sb*-value <0.75 were classified as divergent. Genes with *Sb*-value = 0.75 remained unclassified.

### Microarray data accession number

The microarray data have been deposited in the ArrayExpress database with the series accession number E-TABM-885.

### OG1RF gene prediction

Gene prediction from OG1RF (Genbank ABPI00000000) was conducted with EasyGene 1.2 [32] using model “EF02”, with R cut off value set at 2.

### BLASTN comparison of *E. faecalis* V583 genes versus the OG1RF genome

BLASTN comparison was conducted for *E. faecalis* V583 (Genbank AE016830) against the OG1RF genome (Genbank ABPI00000000) as follows: the annotated V583 genes were blasted (BLASTN) against the entire OG1RF genome, and presence and divergence was predicted based on a score calculated as the number of identical nucleotides divided by the length of the query gene. Genes obtaining a score >0.75 were classified as present.

### Real-time quantitative RT-PCR

Real time quantitative RT-PCR (QPCR) was used to validate the expression levels for selected genes. QPCR was performed on a Rotor-Gene 6000 centrifugal amplification system (Corbett Research). cDNA was synthesized with 1 µg total RNA as template. In addition to the on-column DNase-treatment mentioned above, an off-column DNase treatment was conducted as follows: To each of the RNA preparations, 80 U RNasin, 20 U DNase 1 and 70 µl RDD buffer was added. The reaction was incubated at 37°C for 30 min., and DNase-treated RNA was extracted by performing a phenol:chloroform extraction as follows: 1∶1∶1 (v/v/v) phenol/chloroform/DEPC water was added, before the samples were centrifuged at 10000×g for 1 min. The aqueous layer was transferred to a tube containing 960 µl 96% ethanol and 40 µl 3M NaAc and incubated ON at – 20°C, before the RNA was precipitated by centrifugation at 10000×g for 30 min. at 4°C. RNA was then washed with 70% ethanol, dried by vacuum centrifugation and resuspended in 20 µl RNase-free water. The genes were quantified in triplicate. PCR amplification was performed at an annealing temperature of 60°C with 0.5 µl cDNA in a 25-µl reaction mixture containing 12.5 µl FastStart SYBR green Master (Rox; Roche) and 0.5 µM of each primer. The primers used are shown in Table 2. Upon completing PCR, melting curve analysis was used to determine whether there was detectable primer-dimer contribution to the SYBR green fluorescence measurement of amplified DNA. Differential expression was calculated by the Pfaffl method [111]. 23S was used as a reference.

**Table 2:** QPCR primers used in this study.

Target gene/primer name	Primer sequences (5′ → 3′)	Amplicon size (bp)	Reference
*EF1821*	F: TGA ACC TGT TCA GCC ATC TG	142	This study
	R: CAT CAG ACC TTG GAT GAC GA		
*23S*	F: CCT ATC GGC CTC GGC TTA G		[17]
	R: AGC GAA AGA CAG GTG AGA ATC C		

## Supporting Information

Figure S1 Characterization of growth of E. faecalis MMH594 (black circle), OG1RF (red triangle) and Symbioflor 1 (green square) in 2xYT (stippled lines) and urine (solid lines). The growth curves are represented by colony forming units per millilitre (CFU/ml) on the Y-axis, and hours as indicated on the X-axis. The growth curves correspond to the mean ± STD of two parallels.
(0.10 MB TIF)

Figure S2 Distribution of differentially expressed genes in response to urine by functional classification. Overview of the number of up- and down-regulated genes in MMH594 (grey), OG1RF (purple) and Symbioflor 1 (green) at A) 5 minutes and B) 30 minutes. The functional categories are listed between the two bar-charts.
(0.40 MB TIF)

Figure S3 The effect of urine on the expression of EF1821 (fsrB) in MMH594 an OG1RF as quantified by QPCR.
(5.53 MB TIF)

Table S1 Microarray expression data and comparative genome hybridization of E. faecalis strains MMH594 (M), OG1RF (O) and Symbioflor 1 (S). Differences in gene content were analyzed using comparative genomic hybridization (*): present (1), divergent (0), unclassified (U). Gene expression after 5 (t5) or 30 (t30) minutes of growth in urine is relative to 2xYT. Significantly regulated genes are q<0.01 (bold), and log2-ratio >±0.5). "NA" denotes non-expressed or excluded genes.
(1.10 MB XLS)

Table S2 Gene prediction from OG1RF (Genbank ABPI00000000) conducted with EasyGene 1.2 [32] using model "EF02", with R cut off value at 2. Predicted genes are presented in nucleotide fasta format.
(2.99 MB TXT)

Table S3 BLASTN comparison of E. faecalis V583 genes versus the OG1RF genome. The score was calculated as number of identical nucleotides identified by BLAST divided by query ORF length. ORFs obtaining a score >0.75 were classified as orthologous genes present in the OG1RF genome.
(0.22 MB XLS)

Table S4 Differentially expressed genes with proven or predicted function in various stress responses in *E. faecalis*. Only significant log_2_-ratios are listed.
(0.63 MB DOC)

Table S5 Differentially expressed genes with proven or predicted virulence function in *E. faecalis*. Only significant log_2_-ratios are listed.
(1.20 MB DOC)
