# Supplementary figures and images for: Comparative Genomic Analysis of Pathogenic and Probiotic Enterococcus faecalis Isolates, and Their Transcriptional Responses to Growth in Human Urine
Source: PLoS One. 2010 Aug 31;5(8):e12489. doi: 10.1371/journal.pone.0012489 (PMC2930860; doi:10.1371/journal.pone.0012489)

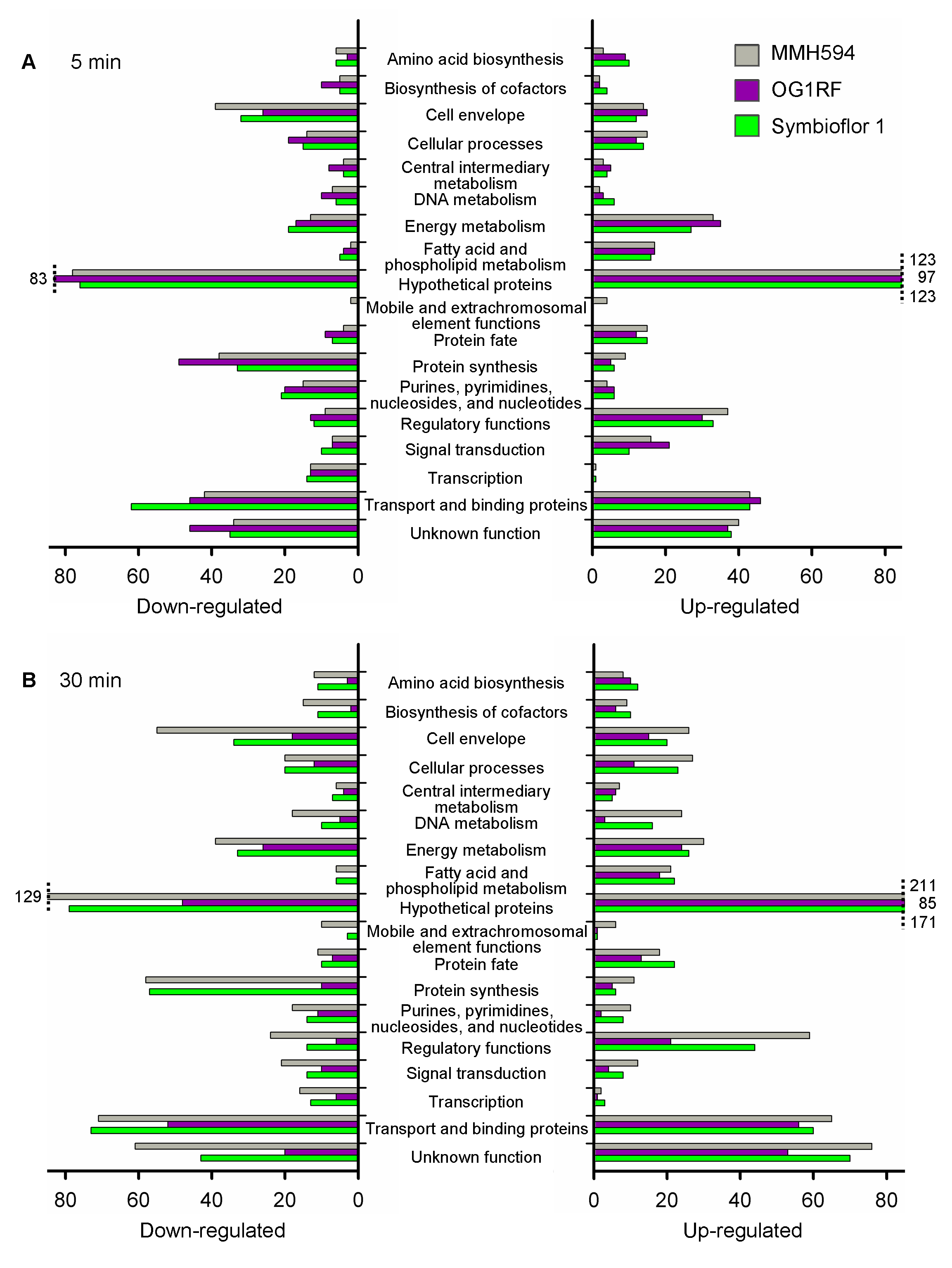

Supplement: Figure S2 — Distribution of differentially expressed genes in response to urine by functional classification. Overview of the number of up- and down-regulated genes in MMH594 (grey), OG1RF (purple) and Symbioflor 1 (green) at A) 5 minutes and B) 30 minutes. The functional categories are listed between the two bar-charts. (0.40 MB TIF) [file pone.0012489.s002.tif]
