# Supplementary material for: Comparative Genomic Analysis of Pathogenic and Probiotic Enterococcus faecalis Isolates, and Their Transcriptional Responses to Growth in Human Urine
Source: PLoS One. 2010 Aug 31;5(8):e12489. doi: 10.1371/journal.pone.0012489 (PMC2930860; doi:10.1371/journal.pone.0012489)
Supplement: Table S4 — Differentially expressed genes with proven or predicted function in various stress responses in E. faecalis. Only significant log2-ratios are listed. (0.63 MB DOC) [file pone.0012489.s007.doc]

**Table S4: Differentially expressed genes with proven or predicted function in various stress responses in *E. faecalis*.** Only significant log_2_-ratios are listed.

| **ORF** | **Other names** | **Gene product** | **Log_2_-ratio*** | | | | | | **Reference** |
| --- | --- | --- | --- | --- | --- | --- | --- | --- | --- |
|  |  |  | **MMH594** | | **OG1RF** | | **Symbioflor 1** | |  |
|  |  |  | *t_5_* | *t_30_* | *t_5_* | *t_30_* | *t_5_* | *t_30_* |  |
| *General stress response* | | |  |  |  |  |  |  |  |
| EF0453 | Gsp65 | OsmC/Ohr family protein | 0.9 | 3.0 | 0.7 | 3.1 | 0.9 | 3.8 | [56,60] |
| EF0770 | Gsp62 | Conserved hypothetical protein | 1.1 | 4.0 | 0.9 | 2.0 | 0.9 | 3.1 | [56,112] |
| EF1058 |  | Universal stress protein family | - | - | - | - | 2.2 | 2.3 | [31] |
| EF1084 |  | Universal stress protein family | - | - | - | - | - | 0.7 | [31] |
| EF1308 | Gsp66 | DnaK protein | 0.8 | - | - | - 1.1 |  | - 0.6 | [56,113] |
| EF1560 | Gsp63 | Hypothetical protein | 1.2 | 3.1 | - | - | - | 1.3 | [56,112] |
| EF1744 |  | General stress protein, putative | 0.6 | 1.8 | - | - | 0.8 | 1.8 | [31] |
| EF1810 |  | General stress protein A (*gspA-1*) | - | 2.1 | - | - | - | - | [31] |
| EF1811 |  | General stress protein A (*gspA-2*) | 1.1 | 4.1 | 1.3 | - | - | 2.7 | [31] |
| EF1982 | Gls33 | Universal stress protein family | 1.1 | 1.6 | 1.4 | - | 1.6 | 2.3 | [31,112,114] |
| EF2633 | Gsp67 | Chaperonin, 60 kDa (*groEL*) | 0.8 | - | - | - 1.1 | - | - | [56,113] |
| EF2797 | Gsp64 | Hypothetical protein | - | - | - | - | 0.6 | 1.1 | [56,112] |
| EF2894 |  | General stress protein 13, putative | - | - | - | - | 0.6 | - | [31] |
| EF3035 |  | Universal stress protein family | - | - | 0.7 | - | 0.5 | - | [31] |
| *Oxidative stress* | |  |  |  |  |  |  |  |  |
| EF0074 |  | Transcriptional regulator, Crp/Fnr family (*ers*) | - | - | - 0.9 | - | - 1.1 | - 0.8 | [64] |
| EF0463 |  | Superoxide dismutase (*sodA*) | 1.0 | 3.5 | - | 2.1 | - | 3.3 | [31] |
| EF1211 |  | NADH peroxidase (*npr*) | 0.7 | 3.0 | 0.8 | 2.2 | 0.5 | 3.7 | [31] |
| EF1338 |  | Thioredoxine reductase (*trxB*) | - | 1.2 | - | 1.4 | - | 1.1 | [31] |
| EF1405 |  | Thioredoxine (*trx*) | - | 1.6 | - |  | - | 1.3 | [115] |
| EF1525 |  | Transcriptional regulator, Fur family (*fur*) | - | - 0.6 | - | - | - | - | [115,116] |
| EF1585 |  | Transcriptional regulator, Fur family (*perR*) |  | 1.3 | - | 0.7 | - | 1.5 | [115,116] |
| EF1586 |  | NADH oxidase (*nox*) | - 1.2 |  | - 1.2 | 0.9 | - 0.8 | 0.7 | [31] |
| EF1597 |  | Catalase (*katA*) | - 0.8 | 2.0 | - 1.2 | - 1.0 | - | 1.1 | [31] |
| EF1681 |  | Peptide methionine-S-sulfoxide reductase (*msrA*) | - | 3.2 | - | - | - | 1.7 | [115] |
| EF2055 |  | Oxidoreductase, pyridine nucleotide-disulfide family | - 0.7 | 1.4 | - 0.8 | 1.5 | -0.9 | 0.8 | [31] |
| EF2417 |  | Transcriptional regulator, Fur family (*zur*) | - | - | - | - | - | 0.6 | [115,116] |
| EF2958 |  | Transcriptional regulator, LysR family (*hypR)* | - | - | - | - | 0.5 | 0.9 | [117,118] |
| EF3164 |  | Peptide methionine-R-sulfoxide reductase (*msrB/ csrA*) | - 0.6 | 4.4 | - | 1.7 | - | 3.3 | [117,119] |
| EF3233 |  | Dps family protein (*dps)* | 0.7 | 2.0 | 0.6 | 1.6 | 1.0 | 2.1 | [31] |
| EF3257 |  | Oxidoreductase, pyridine nucleotide-disulfide family | - | - 1.0 | - | - | - | - 1.6 | [31] |
| EF3270 |  | Glutathione reductase (*gor*) | - | 0.7 | - | 1.1 | 0.5 | 1.5 | [31] |
| *Osmotic stress* | |  |  |  |  |  |  |  |  |
| EF0295 |  | V-type ATPase, subunit J | - | - 0.7 | - | - | - 0.5 | - 1.0 | [31] |
| EF0402 |  | Na+/H+ antiporter | - 0.8 | - | - | - | - | - 0.9 | [31] |
| EF0636 |  | Na+/H+ antiporter | 1.0 | - 4.5 | 1.2 | - 5.3 | - | - 3.8 | [31] |
| *Metal-ion resistance* | |  |  |  |  |  |  |  |  |
| EF0298 |  | Copper-translocating P-type ATPase |  | 2.3 | 1.2 | 2.1 | 1.7 | 3.4 | [31] |
| EF0299 |  | Copper transport protein CopZ | - | 2.3 |  | 2.7 | - | 4.1 | [31] |
| EF0758 |  | Cadmium-translocating P-type ATPase | - | - |  | - | - | 1.0 | [31] |
| EF0871 |  | Cation-transporting ATPase, E1-E2 family | 0.7 | 0.7 | - | - | - | 1.1 | [31] |
| EF0875 |  | Copper-translocating P-type ATPase | 1.7 | 2.2 | 1.4 | 1.0 | 1.5 | 2.0 | [31] |
| EF1268 |  | Cation-transporting ATPase, E1-E2 family | - | 1.2 | - | - | - | 0.6 | [31] |
| EF1352 |  | Magnesium-translocating P-type ATPase | - | - | 1.3 | - 0.7 | 1.5 | - | [31] |
| EF1400 |  | Cadmium-translocating P-type ATPase | - | - 0.8 | - | - | - | - | [31] |
| EF1519 |  | Cation-transporting ATPase, E1-E2 family | - 0.9 | - 1.0 | - | - | - 0.7 | - 0.8 | [31] |
| EF2623 |  | Cadmium-translocating P-type ATPase | - | - 1.3 | - | - 1.6 | 0.6 | - | [31] |
| *Heat shock* |  |  |  |  |  |  |  |  |  |
| EF1306 |  | Heat-inducible transcription repressor HrcA (*hrcA*) | 1.0 | - 0.8 | 0.7 | - 1.1 | 0.8 | - | [31,113] |
| EF1307 |  | Heat shock protein GrpE (*grpE*) | 1.0 | - | - | - 1.2 | - | - | [31,113] |
| EF1646 |  | Heat shock protein HslVU, ATPase subunit (*hslU*) | 1.2 | 1.7 | 0.5 |  | 0.7 | 0.9 | [31,113] |
| EF1647 |  | Heat shock protein HslV (*hvlV*) | 1.5 | 1.7 | 0.9 |  | 1.1 | 0.6 | [31,113] |
| EF2634 |  | Chaperonin, 10 kDa (*groES*) | 0.9 | - | 0.7 | - | - | - 0.7 | [113] |
| *Glucose starvation* | |  |  |  |  |  |  |  |  |
| EF0079 |  | Gls24 protein (*gls24*) | - | - | 0.5 | 3.1 | - | 3.1 | [114,120] |
| EF0080 |  | Gls24 protein (*glsB*) | 0.7 | 4.4 | 0.9 | 3.3 | 0.8 | 3.0 | [114,120] |
| EF0099 | Gls17 | L-serine dehydratase, iron-sulfur-dependent | - | - 3.8 | 1.6 | - 5.0 | 0.8 | - 2.9 | [112,114] |
| EF0106 | Gls14 | Carbamate kinase |  | - 2.2 |  |  | - | - 2.9 | [112,114] |
| EF1359 | Gls40 | Conserved hypothetical protein |  | 1.0 | 0.6 | - | 0.6 | - 0.6 | [112,114] |
| EF1360 | Gls10 | Dihydroxyacetone kinase family protein |  | 0.7 | 0.6 |  | 0.6 | - | [112,114] |
| EF1361 | Gls27 | Dihydroxyacetone kinase family protein | 0.7 | 2.0 | 0.9 | 1.3 | 0.9 | - | [112,114] |
| EF1962 | Gls23 | Triosephosphate isomerase (*tpiA*) | - | - 1.3 | - | - | - | - | [112,114] |
| EF2500 | Gls37 | GcvH family protein | - | - 1.0 | - 0.8 | - | - 0.6 | - 1.0 | [112,114] |
| PAIef0055^a^ |  | Stress-induced protein Gls24 | - | 0.9 | - | - | - | - | [22] |

*Only significant log_2_-ratios are listed. Ratios were calculated as [sample urine]/[sample 2xYT].

^a^ Present in MMH594 only.
