# Supplementary material for: Comparative Genomic Analysis of Pathogenic and Probiotic Enterococcus faecalis Isolates, and Their Transcriptional Responses to Growth in Human Urine
Source: PLoS One. 2010 Aug 31;5(8):e12489. doi: 10.1371/journal.pone.0012489 (PMC2930860; doi:10.1371/journal.pone.0012489)
Supplement: Table S5 — Differentially expressed genes with proven or predicted virulence function in E. faecalis. Only significant log2-ratios are listed. (1.20 MB DOC) [file pone.0012489.s008.doc]

**Table S5: Differentially expressed genes with proven or predicted virulence function in *E. faecalis*.** Only significant log_2_-ratios are listed.

| **ORF** | | **Gene name** | | **Gene product** | | **Log_2_-ratio*** | | | | | | | | | | | | **Reference** |
| --- | --- | --- | --- | --- | --- | --- | --- | --- | --- | --- | --- | --- | --- | --- | --- | --- | --- | --- |
|  | |  | |  | | **MMH594** | | | | **OG1RF** | | | | **Symbioflor 1** | | | |  |
|  | |  | |  | | **t5** | | **t30** | | **t5** | | **t30** | | **t5** | | **t30** | |  |
| EF0031 | |  | | Membrane protein, putative | | - | | 0,6 | | - | | - | | - | | 1,0 | | [31] |
| EF0032 | |  | | Membrane protein, putative | | - | | - | | - | | 0,9 | | - | | 0,7 | | [31] |
| EF0055 | |  | | Adhesion lipoprotein | | -0,7 | | -0,7 | | -1,3 | | - | | - | | -0,8 | | [31] |
| EF0079 | | *gls24* | | Gls24 protein | | - | | - | | 0,5 | | 3,1 | | - | | 3,1 | | [58,59] |
| EF0080 | | *glsB* | | Gls24 protein | | 0,7 | | 4,4 | | 0,9 | | 3,3 | | 0,8 | | 3,0 | | [58,59] |
| EF0089 | |  | | Conserved domain protein | | -0,8 | | -1,3 | | -1,4 | | -2,1 | | -1,0 | | -1,3 | | [31] |
| EF0123 | |  | | Conserved hypothetical protein | | - | | -0,5 | | - | | - | | - | | - | | [31] |
| EF0355 | | *atlB* | | Endolysin, putative | | - | | -1,0 | | - | | - | | - | | - | | [31,84] |
| EF0361 | |  | | Chitinase, family 2 | | 0,9 | | - | | 0,6 | | - | | 0,7 | | - | | [31] |
| EF0362 | |  | | Chitin binding protein, putative | | 1,1 | | - | | 0,8 | | - | | 0,7 | | - | | [31] |
| EF0394 | | *salB* | | Secreted antigen, putative | | -1,5 | | -0,7 | | -3,4 | | - | | -1,1 | | - | | [31,83] |
| EF0463 | | *sodA* | | Superoxide dismutase, Mn | | 1,0 | | 3,5 | | - | | 2,1 | | 0,4 | | 3,3 | | [63] |
| EF0468 | |  | | LemA family protein | | 4,0 | | - | | 5,7 | | - | | 3,8 | | 1,4 | | [31] |
| EF0526 | | *cylL-S* | | CylL-S protein | | - | | 2,2 | | - | | - | | - | | - | | [31] |
| EF0527 | | *cylM* | | CylM protein | | - | | -0,5 | | - | | - | | - | | - | | [31] |
| EF0577 | |  | | Adhesion lipoprotein | | 3,4 | | 4,1 | | - | | - | | - | | - | | [31] |
| EF0645 | |  | | Exfoliative toxin A, putative | | -0,6 | | - | | - | | - | | -0,8 | | - | | [31] |
| EF0680 | |  | | Penicillin-binding protein 2A | | - | | - | | 0,6 | | - | | - | | - | | [31] |
| EF0700 | |  | | Hemolysin | | - | | - | | - | | - | | -0,7 | | - | | [31] |
| EF0746 | |  | | Penicillin-binding protein, putative | | - | | 0,7 | | 1,2 | | - | | 0,8 | | - | | [31] |
| EF0799 | | *atlA* | | Autolysin | | -1,7 | | - | | -1,3 | | - | | -1,9 | | - | | [31,79,84,85] |
| EF0927 | |  | | Sensor histidine kinase | | - | | -0,4 | | 0,6 | | - | | - | | - | | [31] |
| EF0941 | |  | | ABC transporter, ATP-binding | | - | | - | | - | | - | | -0,6 | | - | | [31] |
| EF0944 | |  | | Extracellular protein, putative | | -1,7 | | -1,5 | | -1,6 | | -0,9 | | -2,1 | | -1,8 | | [31] |
| EF0955 | | *bopC* | | Aldose 1-epimerase, putative | | - | | - | | 1,2 | | - | | - | | - | | [77] |
| EF0956 | | *bopB* | | Beta-phosphoglucomutase | | - | | - | | 1,2 | | - | | - | | - | | [77] |
| EF0991 | | *pbpC* | | Penicillin-binding protein C | | -0,6 | | -1,1 | | -1,1 | | -1,2 | | -1,0 | | -1,8 | | [31] |
| EF1032 | | *drrC* | | Daunorubicin resistance protein | | - | | 1,4 | | 1,1 | | 1,2 | | 0,5 | | 1,7 | | [31] |
| EF1038 | |  | | Lipoprotein, putative | | 0,7 | | - | | 1,2 | | 1,7 | | - | | - | | [31] |
| EF1050 | | *etaR* | | DNA-binding response regulator | | - | | - | | -1,0 | | - | | -0,8 | | -1,0 | | [121] |
| EF1051 | | *etaS* | | Sensor histidine kinase | | - | | -0,9 | | -0,7 | | - | | -0,8 | | - | | [121] |
| EF1057 | |  | | Mn2+/Fe2+ transporter | | 4,1 | | 3,9 | | 4,0 | | 4,6 | | 4,0 | | 3,9 | | [31] |
| EF1148 | |  | | Penicillin-binding protein 1A | | - | | -0,4 | | - | | - | | -0,7 | | -0,9 | | [31] |
| EF1340 | |  | | Pheromone cAM373 prec.lipoprot. | | -0,8 | | -1,5 | | -1,6 | | -0,9 | | -0,8 | | -1,1 | | [31] |
| EF1502 | |  | | Beta-lactamase, putative | | -1,2 | | -3,0 | | -0,8 | | -1,6 | | -0,7 | | -1,7 | | [31] |
| EF1603 | | *scrB-1* | | Sucrose-6-phosphate dehydrogenase | | 2,0 | | 3,8 | | 2,3 | | - | | 3,2 | | 4,4 | | [40,41] |
| EF1604 | | *scrR-1* | | Sucrose operon repressor ScrR | | - | | 1,7 | | - | | - | | - | | 3,1 | | [40,41] |
| EF1740 | |  | | Penicillin-binding protein 1B, putative | | - | | -0,5 | | - | | - | | - | | - | | [31] |
| EF1818 | | *gelE* | | Coccolysin | | -0,5 | | - | | - | | - | | - | | - | | [25,31,101] |
| EF1820 | | *fsrC* | | Histidine kinase, putative | | - | | 1,0 | | - | | - | | - | | - | | [25,101] |
| EF1821 | | *fsrB* | | AgrBfs protein | | - | | 1,0 | | - | | - | | - | | - | | [25,101] |
| EF1822 | | *fsrA* | | Response regulator | | 0,7 | | 0,8 | | - | | - | | - | | - | | [25,101] |
| EF2074 | | *efaC* | | ABC transporter | | 2,8 | | 3,0 | | 3,1 | | 3,1 | | 3,7 | | 3,4 | | [54] |
| EF2075 | *efaB* | | ABC transporter | | 2,7 | | 3,5 | | 3,0 | | 3,7 | | 3,6 | | 3,4 | | [54] | |
| EF2076 | *efaA* | | Endocarditis specific antigen | | 2,3 | | 2,9 | | 2,8 | | 3,4 | | 3,0 | | 3,2 | | [31] [54] | |
| EF2093 |  | | Endolysin domain protein | | - | | -0,6 | | - | | - | | - | | - | | [31] | |
| EF2167 |  | | Glycosyl transferase | | -1,1 | | -2,6 | | - | | - | | - | | - | | [31] | |
| EF2170 |  | | Glycosyl transferase | | - | | -1,3 | | - | | - | | - | | - | | [31] | |
| EF2174 |  | | Conserved domain protein | | -0,9 | | - | | - | | - | | -1,0 | | -0,9 | | [31] | |
| EF2176 |  | | Glycosyl transferase | | -0,8 | | -1,0 | | -1,7 | | - | | -1,1 | | -0,5 | | [31] | |
| EF2177 | *epaR* | | Bacterial sugar transferase | | -1,1 | | - | | - | | - | | -0,8 | | -1,6 | | [71,96,97] | |
| EF2178 | *epaQ* | | Membrane protein, putative | | -1,3 | | -1,9 | | -0,9 | | -1,2 | | -0,5 | | -2,1 | | [71,96,97] | |
| EF2179 | *epaP* | | Conserved hypothetical protein | | -2,5 | | -3,6 | | -1,2 | | -0,9 | | -1,0 | | -2,7 | | [71,96,97] | |
| EF2180 | *epaO* | | glycosyl transferase, | | -1,4 | | -1,5 | | -0,8 | | - | | -0,6 | | -1,6 | | [71,96,97] | |
| EF2181 | *epaN* | | Glycosyl transferase | | -0,8 | | -2,1 | | -0,5 | | -0,7 | | -0,9 | | -1,7 | | [71,96,97] | |
| EF2182 | *epaM* | | ABC transporter | | -0,9 | | -1,5 | | - | | - | | -0,9 | | -2,1 | | [71,96,97] | |
| EF2183 | *epaL* | | ABC transporter | | -0,9 | | -1,9 | | - | | - | | -1,1 | | -2,3 | | [71,96,97] | |
| EF2184 | *epaK* | | Hypothetical protein | | -0,7 | | -1,6 | | -1,2 | | - | | -0,8 | | -1,8 | | [71,96,97] | |
| EF2186 |  | | Conserved domain protein | | -1,2 | | -2,9 | | - | | - | | - | | - | | [71,96,97] | |
| EF2188 |  | | Racemase domain protein | | -1,2 | | -1,8 | | - | | - | | - | | - | | [71,96,97] | |
| EF2189 | *epaJ* | | Conserved hypothetical protein | | -1,2 | | -2,2 | | -1,1 | | - | | -0,9 | | -1,4 | | [71,96,97] | |
| EF2190 | *epaI* | | Glycosyl transferase | | -1,1 | | -2,0 | | -0,9 | | - | | -0,6 | | -1,6 | | [71,96,97] | |
| EF2191 | *epaH* | | dTDP-4-dehydrorhamnose reductase | | -1,0 | | -1,5 | | -0,7 | | - | | -0,8 | | -1,5 | | [71,96,97] | |
| EF2192 | *epaG* | | dTDP-glucose 4,6-dehydratase | | -0,7 | | - | | - | | - | | -0,8 | | - | | [71,96,97] | |
| EF2193 | *epaF* | | dTDP-4-dehydrorhamnose | | -0,7 | | - | | -0,7 | | - | | -0,6 | | -0,9 | | [71,96,97] | |
| EF2194 | *epaE* | | Glucose-1-ph thymidylyltransferase | | -0,9 | | -1,1 | | -0,7 | | -0,6 | | -0,9 | | -1,8 | | [71,96,97] | |
| EF2195 | *epaD* | | Glycosyl transferase | | -1,2 | | -1,7 | | - | | -0,5 | | -0,9 | | -1,2 | | [71,96,97] | |
| EF2196 | *epaC* | | Glycosyl transferase | | -1,0 | | - | | - | | - | | -0,5 | | -0,5 | | [71,96,97] | |
| EF2197 | *epaB* | | Glycosyl transferase | | -0,9 | | - | | - | | - | | -0,5 | | -0,5 | | [71,96,97] | |
| EF2198 | *epaA* | | Glycosyl transferase | | -0,8 | | -0,9 | | - | | - | | -0,5 | | -1,0 | | [71,96,97] | |
| EF2202 |  | | TspO protein, putative | | 1,0 | | 2,8 | | - | | - | | - | | 1,7 | | [31] | |
| EF2439 |  | | Undecaprenol kinase, putative | | -1,4 | | -1,3 | | -1,5 | | - | | -1,5 | | -1,8 | | [31] | |
| EF2485 | *cpsK* | | ABC transporter | | - | | -3,1 | | - | | - | | - | | - | | [70,99] | |
| EF2486 | *cpsJ* | | ABC transporter | | - | | -3,1 | | - | | - | | - | | - | | [70,99] | |
| EF2487 | *cpsI* | | UDP-galactopyranose mutase | | - | | -2,7 | | - | | - | | - | | - | | [70,99] | |
| EF2488 | *cpsH* | | Lipoprotein, putative | | - | | -2,1 | | - | | - | | - | | - | | [70,99] | |
| EF2489 | *cpsG* | | MurB family protein | | - | | -2,8 | | - | | - | | - | | - | | [70,99] | |
| EF2490 | *cpsF* | | Conserved hypothetical protein | | - | | -3,1 | | - | | - | | - | | - | | [70,99] | |
| EF2491 | *cpsE* | | Glycosyl transferase | | - | | -2,5 | | - | | - | | - | | - | | [70,99] | |
| EF2492 | *cpsD* | | Glycosyl transferase | | -0,7 | | -2,3 | | - | | - | | - | | - | | [70,99] | |
| EF2493 | *cpsC* | | Teichoic acid biosynth. prot., putative | | - | | -1,0 | | - | | - | | - | | - | | [70,99] | |
| EF2496 |  | | Pheromone cOB1 precursor/lipoprot. | | - | | - | | - | | - | | -0,9 | | - | | [31] | |
| EF2658 |  | | FemAB family protein | | - | | - | | -0,8 | | - | | - | | - | | [31] | |
| EF2680 |  | | ABC transporter | | - | | 0,7 | | - | | - | | - | | - | | [31] | |
| EF2713 |  | | Cell wall surface anchor family protein | | 2,2 | | - | | - | | - | | - | | - | | [31] | |
| EF2751 |  | | Permease protein, putative | | 0,7 | | 1,2 | | - | | - | | - | | 0,8 | | [31] | |
| EF2802 |  | | Endolysin | | - | | - | | - | | - | | - | | -0,6 | | [31] | |
| EF2890 |  | | Glycosyl transferase | | - | | -0,6 | | - | | -1,2 | | - | | - | | [31] | |
| EF2891 | *bgsA* | | Glycosyl transferase | | - | | - | | - | | -1,1 | | - | | - | | [31,122] | |
| EF2929 |  | | Membrane protein, putative | | - | | 0,8 | | - | | - | | -0,5 | | - | | [31] | |
| EF3023 |  | | Polysaccharide lyase, family 8 | | - | | - | | - | | - | | - | | -1,1 | | [31] | |
| EF3056 |  | | Sortase family protein | | - | | 1,1 | | - | | - | | 0,8 | | 1,1 | | [79,80,81] | |
| EF3060 | *salA* | | Secreted lipase, putative | | -3,5 | | -0,7 | | -3,6 | | -1,1 | | -3,1 | | -0,7 | | [31,83] | |
| EF3076 |  | | Cell wall surface anchor family protein | | - | | -0,5 | | - | | - | | - | | - | | [31] | |
| EF3082 |  | | Iron compound ABC transporter | | - | | 2,3 | | - | | 3,4 | | -0,6 | | 1,7 | | [31] | |
| EF3083 |  | | Iron compound ABC transporter | | - | | 2,3 | | - | | 3,3 | | - | | 1,5 | | [31] | |
| EF3084 |  | | Iron compound ABC transporter | | -0,7 | | 2,7 | | - | | 4,0 | | -0,6 | | 1,7 | | [31] | |
| EF3085 |  | | Iron compound ABC transporter | | - | | 2,0 | | - | | 2,7 | | - | | 2,5 | | [31] | |
| EF3106 |  | | Peptide ABC transporter | | 0,6 | | 5,1 | | 0,8 | | 3,4 | | - | | - | | [31] | |
| EF3129 |  | | D-alanyl-D-alanine carboxypeptidase | | -0,6 | | -0,8 | | -0,7 | | -0,7 | | - | | - | | [31] | |
| EF3183 |  | | Cell wall surface anchor family protein | | -0,7 | | -1,1 | | - | | - | | - | | - | | [31] | |
| EF3187 |  | | Cell wall surface anchor family protein | | -0,8 | | -0,9 | | - | | - | | - | | - | | [31] | |
| EF3191 |  | | Lipase, putative | | - | | 0,6 | | - | | - | | - | | 0,8 | | [31] | |
| EF3198 |  | | Lipoprotein, YaeC family | | - | | 0,7 | | - | | 0,7 | | - | | 1,5 | | [31] | |
| EF3206 |  | | Adhesion lipoprotein | | - | | - | | - | | 1,1 | | - | | - | | [31] | |
| EF3245 |  | | Cell-envelope ass. acid phosphatase | | - | | -0,5 | | - | | - | | - | | - | | [31] | |
| EF3256 |  | | Pheromone cAD1 precursor lipoprot. | | -0,6 | | -2,4 | | - | | - | | - | | -0,6 | | [31] | |
| EF3314 |  | | Cell wall surface anchor family protein | | - | | - | | - | | - | | 0,9 | | 3,5 | | [31,82] | |
| EF3331 |  | | Pheromone cCF10 percursor/lipoprot. | | - | | -1,5 | | - | | -1,1 | | - | | -1,4 | | [31] | |
| PAIef0049^a^ | *cyl I* | | CylI protein | | - | | -0,9 | | - | | - | | - | | - | | [6,7,100] | |

*Only significant log_2_-ratios are listed. Ratios were calculated as [sample urine]/[sample 2xYT].

^a^ Present in MMH594 only.
